# Two doses of the SARS-CoV-2 BNT162b2 vaccine enhance antibody responses to variants in individuals with prior SARS-CoV-2 infection

**DOI:** 10.1126/scitranslmed.abj0847

**Published:** 2021-09-01

**Authors:** Richard A. Urbanowicz, Theocharis Tsoleridis, Hannah J. Jackson, Lola Cusin, Joshua D. Duncan, Joseph G. Chappell, Alexander W. Tarr, Jessica Nightingale, Alan R. Norrish, Adeel Ikram, Ben Marson, Simon J. Craxford, Anthony Kelly, Guruprasad P. Aithal, Amrita Vijay, Patrick J. Tighe, Jonathan K. Ball, Ana M. Valdes, Benjamin J. Ollivere

**Affiliations:** 1 Wolfson Centre for Global Virus Research, University of Nottingham, A Floor, West Block, Queen's Medical Centre, Derby Road, Nottingham NG7 2UH, UK.; 2 NIHR Nottingham Biomedical Research Centre, Nottingham University Hospitals NHS Trust and University of Nottingham, Queen's Medical Centre, Derby Road, Nottingham NG7 2UH, UK.; 3 School of Life Sciences, University of Nottingham, A Floor, West Block, Queen's Medical Centre, Derby Road, Nottingham NG7 2UH, UK.; 4 Department of Infection Biology and Microbiomes, Institute of Infection, Veterinary and Ecological Sciences, University of Liverpool, Liverpool Science Park IC2, 146 Brownlow Hill, Liverpool L3 5RF, UK.; 5 School of Life Sciences, University of Nottingham, Life Sciences Building, University Park Campus, Nottingham NG7 2RD, UK.; 6 Injury, Inflammation & Recovery Sciences, School of Medicine, University of Nottingham, C Floor, West Block, Queen's Medical Centre, Derby Road, Nottingham NG7 2UH, UK.; 7 Trauma and Orthopaedics, University Hospitals Nottingham, C Floor, West Block, Queen's Medical Centre, Derby Road, Nottingham NG7 2UH, UK.

## Abstract

Understanding the impact of prior infection with severe acute respiratory syndrome coronavirus 2 (SARS-CoV-2) on the response to vaccination is a priority for responding to the coronavirus disease 2019 (COVID-19) pandemic. In particular, it is necessary to understand how prior infection plus vaccination can modulate immune responses against variants of concern. To address this, we sampled 20 individuals with and 25 individuals without confirmed previous SARS-CoV-2 infection from a large cohort of health care workers followed serologically since April 2020. All 45 individuals had received two doses of the Pfizer-BioNTech BNT162b2 vaccine with a delayed booster at 10 weeks. Absolute and neutralizing antibody titers against wild-type SARS-CoV-2 and variants were measured using enzyme immunoassays and pseudotype neutralization assays. We observed antibody reactivity against lineage A, B.1.351, and P.1 variants with increasing antigenic exposure, through either vaccination or natural infection. This improvement was further confirmed in neutralization assays using fixed dilutions of serum samples. The impact of antigenic exposure was more evident in enzyme immunoassays measuring SARS-CoV-2 spike protein–specific IgG antibody concentrations. Our data show that multiple exposures to SARS-CoV-2 spike protein in the context of a delayed booster expand the neutralizing breadth of the antibody response to neutralization-resistant SARS-CoV-2 variants. This suggests that additional vaccine boosts may be beneficial in improving immune responses against future SARS-CoV-2 variants of concern.

## Protection from infection

Antibody responses against severe acute respiratory syndrome coronavirus 2 (SARS-CoV-2) are elicited by both infection and vaccination. However, the extent to which prior infection influences the response to vaccination is unclear. Here, Urbanowicz *et al.* evaluated antibody reactivity and neutralization potency in serum samples collected from individuals who received the BNT162b2 SARS-CoV-2 vaccine with or without a prior history of infection. The authors found that, regardless of prior infection status, vaccination elicited antibodies that bound to SARS-CoV-2 spike proteins, including spike proteins from variants of concern. However, prior infection further enhanced anti-spike protein antibody responses against variants. These findings suggest that repeated exposure to antigen, through either infection or booster vaccinations, augments immune responses to SARS-CoV-2.

## Teaser

Antibody responses to SARS-CoV-2 variants increase after two doses of BNT162b2 vaccine in both previously infected and naïve individuals.

## INTRODUCTION

Clinical trials of severe acute respiratory syndrome coronavirus 2 (SARS-CoV-2) vaccines have collectively involved thousands of participants, providing evidence to support expedient and widespread vaccination globally (*1*–*4*). In the United Kingdom, the first vaccine to be given emergency authorization was the Pfizer-BioNTech stabilized spike-based mRNA vaccine, now marketed as BNT162b2. The vaccines developed to this point are proving to be effective in reducing the infection rate and morbidity and mortality associated with SARS-CoV-2 infection (*2*, *5*, *6*). However, there are concerns about the impact of antigenic viral variants that have recently emerged with spike protein variations, presumably in the face of suboptimal immunity in areas of high virus circulation (*7*). One of these variants, first reported in South Africa (*8*) and known as 501Y.V2 (lineage B.1.351) or the β variant, has been shown to effectively escape from neutralization by convalescent serum samples, monoclonal antibodies, and vaccine-elicited serum samples (*9*–*12*), and poor neutralization may lead to vaccine breakthrough (*13*). Given the potential for antigenic variation to lead to vaccine escape, consideration is being given to provide additional boosts, either with the vaccines containing the original lineage A (Wuhan) viral spike or with antigens matched to emerging variants (*14*). Although increased antigenic exposure is expected to elevate antibody titer and potentially expand the epitope repertoire, previous studies of other respiratory viruses, for example, influenza A, have shown limits to the extent to which repeated antigenic exposure can boost antibody concentrations (*15*).

To investigate the potential for increased antigenic exposure to produce antibody-mediated immunity with increased neutralizing potency against SARS-CoV-2 variants, we analyzed serum samples obtained from a prospective, longitudinal cohort of health care workers (HCWs) collected at regular intervals through the period of pandemic. We analyzed serum samples collected after first and second vaccine doses from individuals previously infected with SARS-CoV-2 and seronegative individuals for the presence of spike-reactive or virus-neutralizing antibodies against lineage A and lineage B.1.351 virus. We also performed neutralization assays against P.1 (γ) variant pseudotypes using available post-boost serum samples.

## RESULTS

### Repeated SARS-CoV-2 antigenic exposure, through vaccination or natural infection, results in increased and more broadly reactive spike-specific antibody responses

**Fig. 1.**
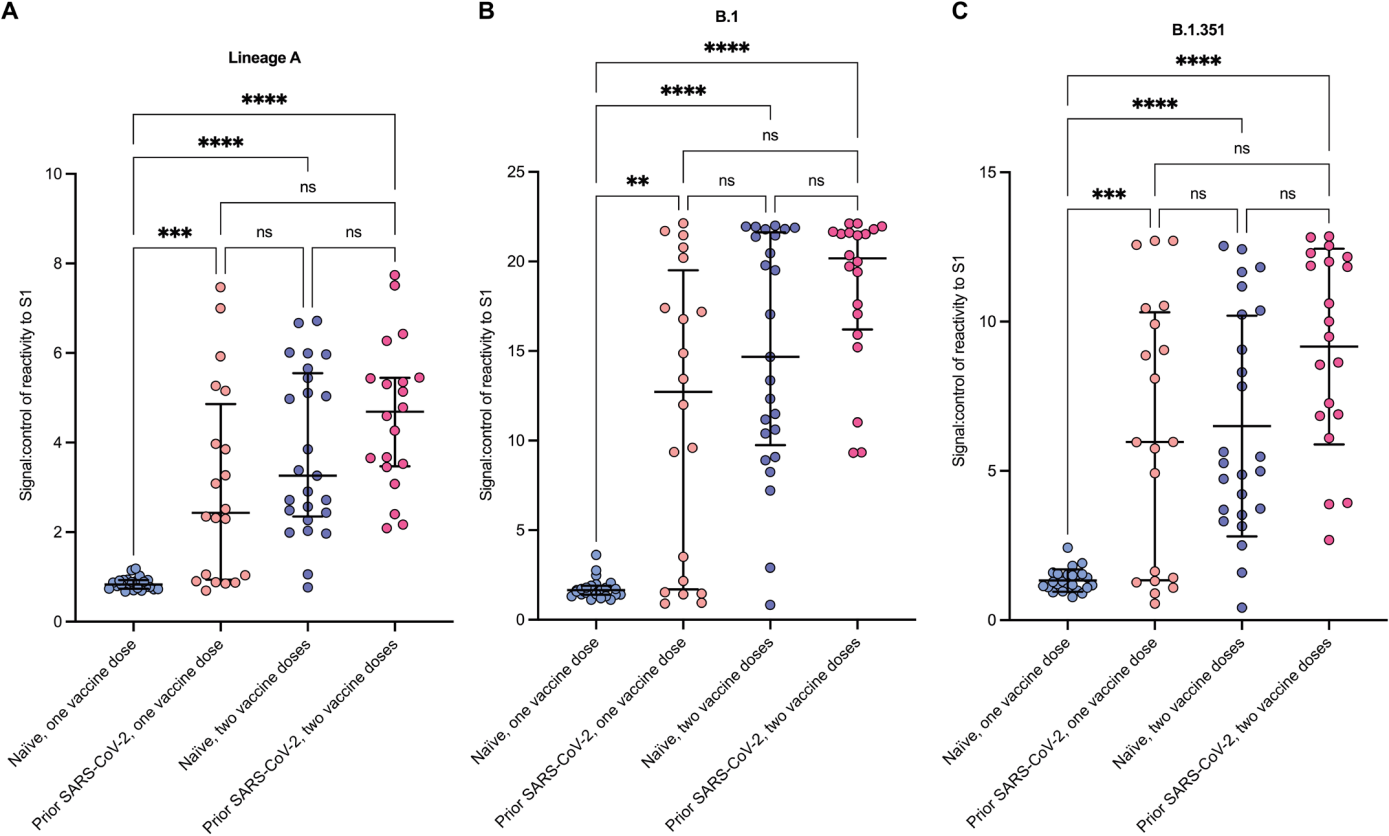
Multiple exposures increase antibody reactivity to SARS-CoV-2 variants. (**A** to **C**) Ratio of reactivity signal to control (Signal:control) of lineage A S1 (A), B.1 S1 (B), and B.1.351 S1 (C) at a 1:600 serum dilution. The number of vaccine doses and infection history is indicated. Plots show median and interquartile ranges (IQRs). Differences between groups were analyzed using a Kruskal-Wallis test with Dunn’s multiple comparisons correction. **P < 0.01; ***P < 0.001; ****P < 0.0001; ns, nonsignificant.

The PANTHER (Pandemic Tracking of Healthcare workers) vaccine cohort consists of frontline HCWs, of which 18% had serological or polymerase chain reaction (PCR)–confirmed past SARS-CoV-2 infection before vaccination (*16*, *17*). Because of occupational risk, and to reduce nosocomial transmission in hospitals, a subset of these people was included in the first-priority group for coronavirus disease 2019 (COVID-19) vaccination. For this study, we identified a total of 45 individuals who had received both doses of the BNT162b2 vaccine with complete serology and prioritized for vaccination and divided them into two groups: those with past infection and those with no evidence of past infection (table S1). The two groups did not differ in terms of age or body mass index (fig. S1), although more females (*n* = 36) than males (*n* = 9) were recruited into the study cohort. Before assessing the S1-specific antibody response, we validated our in-house S1-specific antibody assay against the commercially available assay (Roche) and showed good correlation between the two assays (fig. S2). We then went on to measure the S1-specific antibody response, using the validated in-house enzyme immunoassay, in serum samples obtained 2 days before and then at least 2 weeks after the second (booster) vaccine dose. After the first vaccine dose, concentrations of antibodies specific to the lineage A (Fig. 1A) and lineage B.1 (Fig. 1B) S1 proteins were significantly higher in individuals with evidence of prior infection compared to those who were uninfected (median absorbance ratio of 2.43 versus 0.83; *P* = 0.0008 and 12.72 versus 1.65; *P* = 0.0045, respectively). Antibody concentrations in individuals with past infection and a single dose of vaccine were comparable to uninfected individuals after two doses of vaccine. The booster dose significantly increased both lineage A–specific and lineage B.1–specific antibody concentrations in both groups (lineage A, no infection, two doses versus 1 dose, *P* < 0.0001; B1, no infection, two doses versus one dose, *P* < 0.001). Although most individuals showed an increase in S1 antibody concentrations to all variant S1 proteins, before and after plots showed that, in a small proportion (5 of 45 or 11%) of individuals, the antibody concentration decreased after boosting (fig. S3). One individual who had no prior exposure to SARS-CoV-2 generated a potent antibody response from a low baseline value after a single dose of vaccine comparable to that seen in individuals with prior infection. The before and after plots also showed that antibody reactivity before and after boost to the S2 subunit of spike was relatively low, when compared to S1, in most serum samples (median absorbance ratio of 1.157 versus 1.779 after boost), although they did show a significant increase in the individuals who did not have a natural infection (*P* = 0.01205). As expected, reactivity to nucleocapsid (0.815 versus 0.787 after boost) was also low (fig. S3).

A similar analysis against S1 protein representing the neutralization-resistant B.1.351 S1 protein revealed more variability in recognition after multiple exposures to S1 antigen (*18*), although, in general, increasing antigenic exposure through either natural infection or vaccination resulted in increased reactivity. The average reactivity in uninfected individuals after a single dose of vaccine was relatively low initially but increased significantly after the vaccine boost (median absorbance ratio of 1.24 and 5.27, respectively, *P* < 0.0001; Fig. 1C). A single vaccine dose in previously infected people elicited significantly higher antibody reactivity (median absorbance ratio = 5.97, *P* = 0.0006) than the prime-dose response observed in uninfected individuals. Last, we revalidated the in-house assay against the Roche commercial spike antibody assay using samples collected after vaccine boost, and again, there was a strong correlation between the two assays (fig. S2).

### Increasing antigenic exposure preferentially increases targeting of epitopes outside of the receptor binding domain

**Fig. 2.**
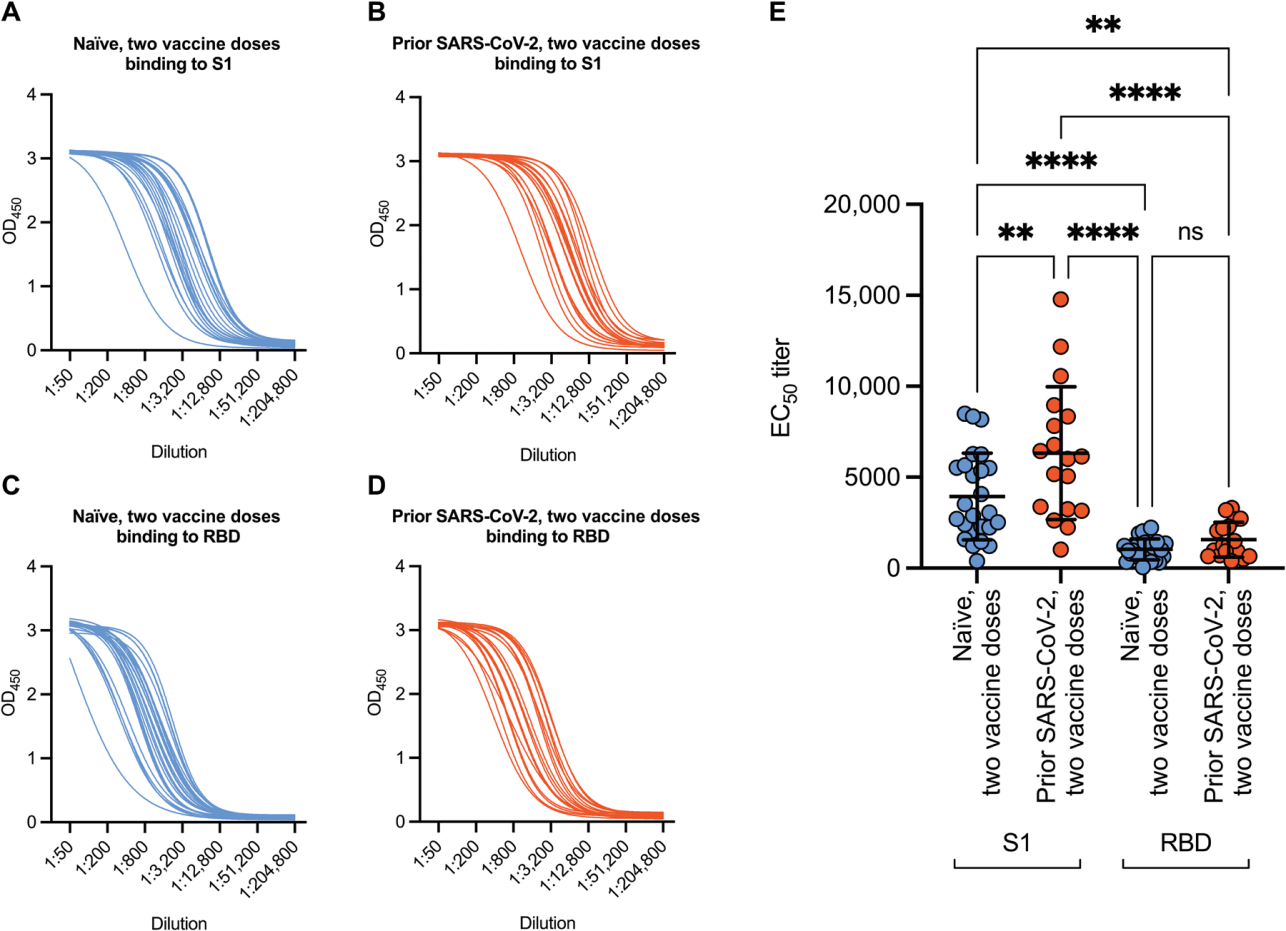
Prior history of SARS-CoV-2 infection increases binding to the S1 region of lineage A SARS-CoV-2 relative to those without prior history of infection. (**A** and **B**) Binding of antibodies to lineage A S1 was measured in serum samples isolated from participants after two doses of vaccine without prior infection (A) or with prior infection (B). OD_450_, optical density at 450 nm. (**C** and **D**) Binding to the lineage A RBD was also evaluated in serum samples collected after two doses of vaccine without prior infection (C) or with prior infection (D). (**E**) EC_50_ titers are shown based on prior history of infection and binding. Because of sample constraints, the two doses with infection group *n* = 18. The plot in (E) shows mean ± SD. Differences between groups in (E) were evaluated using one-way ANOVA with Tukey’s multiple comparisons test. **P < 0.01; ****P < 0.0001.

To provide insight into the type of antibody response generated by multiple exposures, we titrated serum samples from both groups after two doses of vaccine and evaluated their reactivity against lineage A S1 and receptor binding domain (RBD) of the same strain (Fig. 2, A to D). The median effective concentration (EC_50_) titers against the S1 region of the spike protein were significantly higher in individuals with two vaccine doses and natural infection compared to individuals who had two doses of the vaccine and no prior history of infection (6328 versus 3936, *P* = 0.0034; Fig. 2E). The EC_50_ against RBD was lower than against full S1 in both cohorts, but no difference was observed between two doses with and without natural infection [1568 versus 1039, nonsignificant (ns); Fig. 2E]. These data suggest that the response elicited by multiple exposures to spike protein is being directed toward epitopes outside of the RBD.

### Prior infection and vaccine boosting increases neutralizing antibody titer

**Fig. 3.**
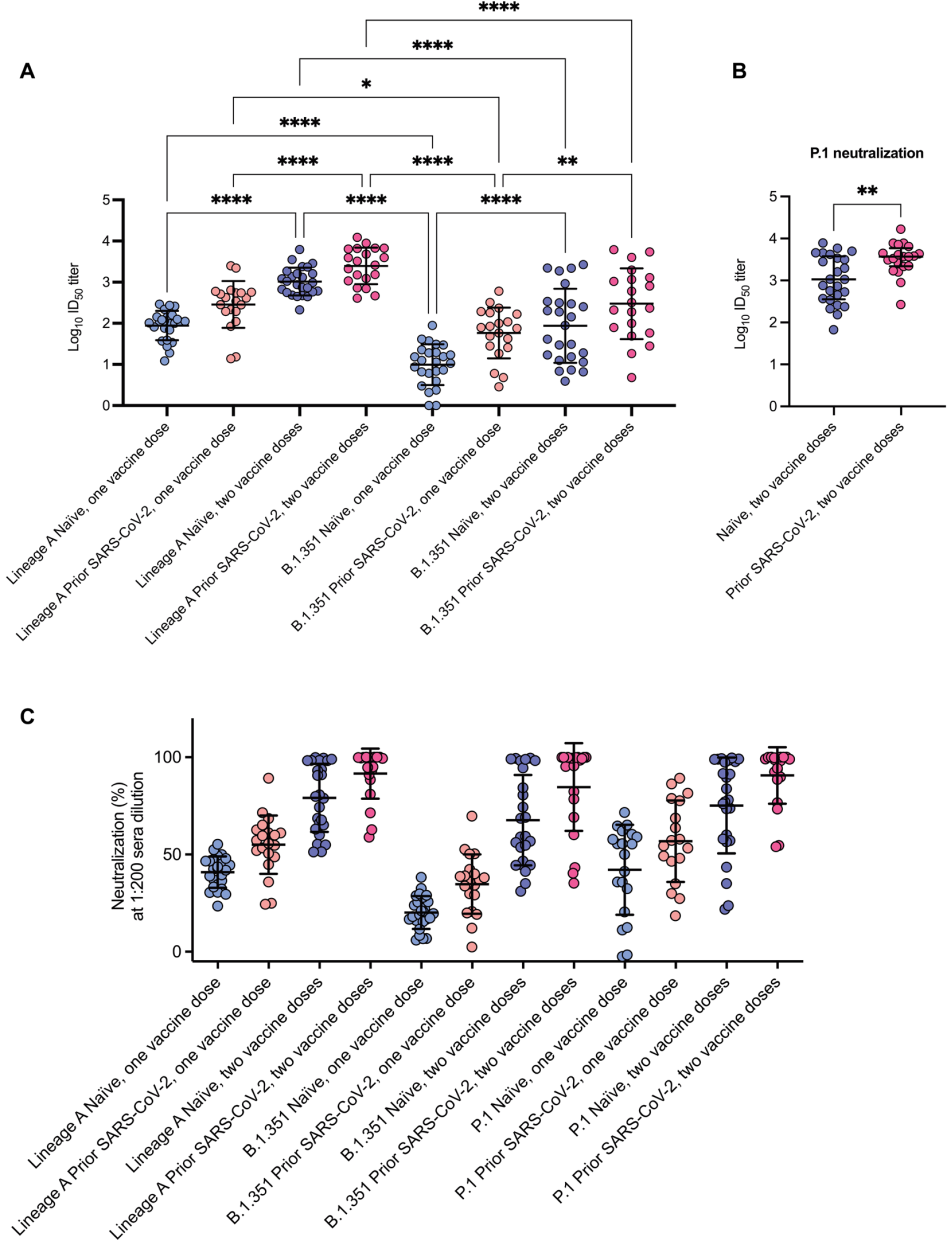
Neutralization potency of SARS-CoV-2–specific antibodies increases with exposure to spike protein through infection or vaccination. (**A**) Log_10_ ID_50_ of serum samples specific to lineage A or B.1.351 pseudoviruses. (**B**) Log_10_ ID_50_ of serum against P.1 variant pseudovirus for samples collected after two doses with (*n* = 18) and without (*n* = 20) prior history of SARS-CoV-2 infection. Sample sizes are smaller due to sample constraints. (**C**) Percent neutralization of serum samples diluted 1:200 for lineage A, B.1.351, and P.1 pseudoviruses. Samples are shown by number and type of exposure. Data are presented as mean ± SD (A and C) or median and IQR (B) due to not passing the D’Agostino and Pearson normality test. Differences between groups in (A) and (C) were evaluated using two-way ANOVA with Tukey’s multiple comparisons test. Differences between groups in (B) were evaluated using two-tailed Mann-Whitney test. *P < 0.05; **P < 0.01; ****P < 0.0001. Significance values for (C) are shown in table S2.

Having established that past infection and vaccine boosting increased S1-specific antibody concentrations, we used pseudotyped lentiviruses expressing either lineage A or B.1.351 spike protein to determine virus neutralization curves (fig. S4) after each dose of vaccine in the two groups and then compared the resulting median infective dose (ID_50_) values (Fig. 3A). In the analyses using lineage A pseudoviruses, the average serum ID_50_ neutralizing titers after a single dose of vaccine were higher in those who had a past infection with SARS-CoV-2 compared to those without previous infection (535.4 versus 114.7, *P* = 0.0008; Fig. 3A). Similarly, mean reciprocal neutralizing antibody titers were higher in uninfected individuals after two vaccine doses compared to previously infected individuals (1405 versus 535.4, *P* = 0.0002) who had received a single dose. Vaccine boosting increased neutralizing antibody titers in both groups when compared to titers obtained after a single dose of vaccine. There was a 12-fold change in the mean reciprocal titer for uninfected individuals and a 7-fold increase for samples from previously infected individuals, and both increases were statistically significant (*P* < 0.0001 for both).

We assessed the impact of antigenic variation using pseudoviruses bearing the lineage B.1.351 spike protein (Fig. 3A). Repeated antigenic exposure resulted in an increase in neutralization of the B.1.351 variant. For example, in previously infected individuals, the mean ID_50_ neutralizing titer increased from about 1:60 after the initial dose of vaccine to 1:300 after the booster dose (*P* = 0.0019). Neutralizing titers for serum samples were consistently greater against pseudoviruses complemented with the lineage A variant spike protein as compared to the neutralization-resistant B.1.351 spike variant.

To determine whether the boosting effect of prior exposure was a general phenomenon, we determined the neutralizing potency of post-boost serum samples against pseudoviruses bearing a spike protein from a different variant of concern, the P.1 or γ variant first identified in Brazil. Comparison of the average reciprocal serum dilution ID_50_ values showed significantly higher concentrations of neutralizing antibodies in serum samples obtained from individuals with prior SARS-CoV-2 infection compared to those with no known previous infection (3703 versus 1068, *P* = 0.0074; Fig. 3B).

We then established the impact of antigenic variation on predicted vaccine effectiveness in the entire cohort by measuring pseudotype neutralization using a fixed 1:200 dilution of serum. We selected a 1:200 dilution because previous SARS-CoV-2 challenge studies in nonhuman primates have shown that serum samples yielding an in vitro IC_50_ neutralization titer of at least 1:200 are protective (*19*). After a single dose of BNT162b2, the degree of neutralization in individuals without previous infection was significantly higher against the lineage A spike virus as compared to B.1.351 (40.9 versus 20.2%, *P* < 0.0001). However, the average values were all less than 50% neutralization (Fig. 3C and table S2). Neutralizing antibodies against both variants increased with increasing antigenic exposure through either natural infection or vaccination. After the final vaccine boost, the ability of serum samples to neutralize virus was significantly lower for the B.1.351 variant compared to lineage A (*P* = 0.0345) and P.1 (*P* = 0.0302). However, no difference was observed after vaccine boost in previously infected individuals, with the average neutralization (lineage A 91.6% and B.1.351 84.7%) greater than 50% for both variants with all achieving effective neutralization against lineage A and 85% against B.1.351. Similarly, the average percentage neutralization observed for a fixed dilution of post-boost serum samples against the P.1 variant was 82% and 49% in previously infected versus SARS-CoV-2 naïve individuals, respectively (*P* < 0.0001) (Fig. 3C and table S2). Last, we observed a positive correlation between total S1 antibody concentrations and neutralization titers (lineage A: *r* = 0.5583, *P* < 0.0001; B.1.351: *r* = 0.4452, *P* < 0.0001) (fig. S5).

## DISCUSSION

Using a well-characterized HCW cohort, we have been able to quantify the effect of a booster vaccination strategy on neutralizing or total immunoglobulin G (IgG) antibody responses to SARS-CoV-2 and variants of concern. We focused on IgG antibody concentrations because these are the predominant antibody class induced by intramuscular immunization and are associated with long-term protective immunity (*20*). We measured antibody responses in serum samples isolated from naïve individuals and individuals with prior history of SARS-CoV-2 infection at baseline and after vaccination with first and second doses. Although previous studies have shown that antibody responses in previously exposed individuals are greater after a single dose of a COVID-19 vaccine (*21*–*23*), this study shows that the trend toward higher neutralizing antibody frequency in previously infected people continues after the second dose. Our results demonstrate that repeated antigenic exposure increases antibody and neutralizing responses to lineage A, B.1, B.1.351, and P.1 variants, suggesting that vaccination with boosters may be effective in combating variants of concern.

Vaccine effectiveness against neutralization-resistant SARS-CoV-2 variants, such as the B.1.351 variant, is of major public health concern (*10*, *24*) [also reviewed in (*25*)]. This study confirms the neutralization-resistant phenotype of the B.1.351 variant. After one dose of vaccine in individuals with or without prior infection, spike protein–specific IgG and neutralizing antibody concentrations were lower against the B.1.351 variant than lineage A strains. However, increasing antigenic exposure in humans through natural infection followed by a two-dose BNT162b2 vaccination schedule increased IgG concentration and neutralization of the B.1.351 and P.1 variants to values comparable to the lineage A virus. Further characterization of the IgG response suggested that multiple antigenic exposure increases reactivity to epitopes outside of the RBD. This might contribute to the increased cross-reactivity of the antibody response and increased neutralizing activity. However, the relative contribution of repertoire expansion versus increased antibody affinity to increased antibody reactivity is unclear. Reactivity to the S2 region after vaccine boost showed only modest increases compared to S1, especially in individuals without prior exposure to SARS-CoV-2. Previous analysis of BNT162b2 antibody responses has shown that this vaccine elicits measurable S2-specific responses (*26*). Thus, it is unclear why robust S2 responses were not evident in all of our vaccine recipients. Future characterization of S1 and S2 neutralizing determinants, together with analysis of antibody affinity and avidity, will provide a better understanding of protective antibody responses and provide important insights for future vaccine development.

Recent studies have evaluated the durability of anti–SARS-CoV-2 neutralizing antibody titers through longitudinal sampling and modeling of phase 1 and 2 clinical data to estimate thresholds for protection against infection and against severe COVID-19. Independent of these specific thresholds, a lower degree of initial neutralization may result in a shorter time span before the titers are below the protective threshold. In the case of a 108-day half-life for titers, a 25% lower neutralizing titer for a given variant would mean 44.8 fewer days above a given protective threshold, which may be relevant in terms of public health in periods when viral transmission is high.

To evaluate virus neutralization, we used a lentivirus-based pseudovirus assay rather than live virus assays. This was chosen because of the superior tractability of the pseudotype assay compared to live virus neutralization assays. These assays are also less susceptible to cell culture adaptation that is frequently encountered during virus passage. In direct comparisons, the pseudotype assay was shown to produce data that were entirely comparable to more laborious live virus assays (*27*).

Our study has several limitations. Prior exposure in our cohort involves only exposure to the lineage A or B variants (*28*, *29*), and natural infection by more antigenically diverse strains may skew the resultant immune potency and breadth. Unfortunately, we had too few positive PCR samples to try to elucidate any link between immunity and infecting lineage. Up to the start of the vaccination program, Nottingham and the surrounding region recorded a range of B.1-derived sublineages, with expansion of B.1.1.7 (α variant) occurring from January 2021. We did not include B.1.1.7 in our analysis because previous studies had shown that neutralizing antibody responses and vaccine effectiveness to this variant were similar to lineage A (*30*, *31*). Instead, we focused on major variants of concern that had previously been shown to be resistant to neutralizing antibody responses (B.1.351 and P.1). However, other variants, such as the recently described B.1.617.2 (δ variant first recorded India), might respond differently. Therefore, it will be important to further validate the effectiveness of vaccine-elicited serum on a variety of emerging variants of concern. For the antibody binding analysis, we relied on the use of recombinant protein from a commercial source that did not have the P.1 variant available. To ensure data comparability, we did not attempt to source the P.1 variant from an alternative supplier or to produce it in-house. A further limitation is that we have not been able to address the links between neutralization and T cell or B cell responses. Our analyses focused on predominantly female HCWs rather than more vulnerable older populations, and our cohort received only one of several vaccines being rolled out globally. Last, the U.K. vaccination policy at the time of our study dictated an extended interval between prime and boost vaccination of between 10 and 12 weeks. Therefore, it will be important to assess whether this affected immune response in comparison to the widely adopted 3- to 4-week schedule. Related to this, a recent study has indicated that increased antibody responses are associated with the extended dosing interval in those aged over 80 years (*32*). Despite the caveats outlined above, our data provide insight into the impact of multiple antigenic exposures and its effect on increasing antibody potency and breadth, and we expect this to be recapitulated across different vaccines and across people with different vulnerabilities. Certainly, the extensive clinical trial data and ensuing data captured during vaccine rollout suggest that approved vaccines have similar performance and that the response in different groups of recipients is also similar (*4*, *11*, *33*, *34*).

Previous studies have shown that a single-dose vaccine can improve antibody titers and neutralizing potency in previously infected individuals (*35*–*39*). However, this study further explores the effects of prior infection on cross-reactive immunity induced by a complete, two-dose COVID-19 vaccine schedule with administration of the first and second doses separated by 10 weeks. Our results, therefore, capture the effect of a booster dose on neutralization after antibody titers have started to wane [around 30% assuming that they peak at 2 weeks and have a half-life of 108 days (*40*)]. Our data provide compelling evidence that neutralizing potency and breadth is further increased after a vaccine boost, and this effect is even greater if the vaccine recipient has had prior infection with SARS-CoV-2. This indicates that repeated antigenic exposure improves immunity, and in our cohort, there was no evidence for an apparent antibody ceiling, as has been reported previously for influenza A (*15*). This supports the notion that vaccine effectiveness, even against emerging neutralization-resistant variants, may be improved after an additional vaccine boost even if that vaccine boost contained the original lineage A spike protein.

## MATERIALS AND METHODS

### Study design

The PANTHER study is a longitudinal cohort study launched in April 2020 to follow up seropositivity to SARS-CoV-2 among HCWs in Nottingham for 10 weeks. Further serological follow-up was performed in autumn 2020. The cohort is described in detail in the online published protocol (*17*) and in (*16*).

In January 2021, a subset of 45 participants from the above cohort who had already had the first dose of the BNT162b2 vaccine was enrolled and asked to give blood using a blood microsampling device ahead of their booster dose. They were then invited to attend a phlebotomy appointment between 14 and 21 days after their booster dose when a venous sample with serum collection was performed. Samples were collected and stored under a Human Tissue Authority license in the Nottingham Tissue Bank (license number 11035). The study protocol was approved by North West–Greater Manchester South Research Ethics Committee (reference 20/NW/0395). The objective of the present study was to assess differences in serological responses after vaccination among previously infected and infection-naïve individuals.

Participants who had taken part in the previous serological surveillance study were invited to take part in the post-vaccination study, and the first 20 previously infected with SARS-CoV-2 (as shown from their seropositivity status during 2020) and 25 individuals without prior infection were included in the present study. No randomization was performed. Samples were blinded for experiments and unblinded at the point of data assessment. All assays were performed in duplicate (serology) or triplicate (neutralization).

The sample size of *n* = 45 pre-boost versus post-boost vaccination was powered (80% power with *P* < 0.05) to detect differences of 0.43 SD in antibody or neutralization titers between the first and second vaccination doses. For the comparison between infection-naïve and previously infected individuals, the study was powered to detect (80% power *P* < 0.05) differences of 0.87 SDs between groups, which was considered sufficient given that differences of over one order of magnitude in anti-RBD titers after the first vaccination dose (at least 2 SDs) between infection-naïve and preinfected individuals have been reported in a similar setting (*41*).

### Patient samples

We have leveraged the PANTHER study (ISCTRN: 15677965 ethics approved 27/10/2020 North West–Greater Manchester South Research Ethics Committee CPMS 47035, IRAS 285436), a longitudinal prospective observational study of HCWs in the East Midlands who underwent weekly quantitative serology testing from 20 April to 10 July 2020. This testing included quantitative measurements of IgG, IgM, and IgA, neutralization assays, and symptomatology (*17*). A predefined nested substudy to assess the effect of booster dose on antibody reactivity or neutralization to lineage A and two variants (B.1 and B.1.351) was carried out using samples isolated from 45 HCWs [36 women and 9 men, aged 47.5 years (SD = 11.4)] from the PANTHER cohort who had paired pre- and post-booster dose serology, 20 of whom had confirmed SARS-CoV-2 infection before the start of vaccination. Of those with previous SARS-CoV-2 exposure, 8 were diagnosed by PCR, whereas the rest were identified by serological testing in either the second (12 individuals), third (4 individuals), or final quarters (4 individuals) of 2020 (table S1). All participants had two doses of the BNT162b2 vaccine between December 2020 and February 2021 and a booster dose between late February and mid-March 2021. The booster immunization was administered between 10 and 12 weeks after the first vaccine, in accordance with current U.K. vaccine policy (which differs from the schedule of 3 to 4 weeks adopted in other countries). Baseline serum samples were taken and analyzed before the vaccine program commenced. Then, further samples were taken and analyzed on average 68 days after the first vaccine dose (and 2 days before the boost) and 18 to 19 days after the boost for both groups of volunteers (table S1).

### Spike-specific enzyme immunoassay

His-tagged, human embryonic kidney (HEK) 293–expressed spike S1 variants lineage A (Wuhan), B.1 variant, lineage A spike S2 domain, and baculovirus-expressed nucleocapsid were produced by SinoBiologicals (Stratech Scientific UK). His-tagged, HEK293-expressed recombinant B.1.351 variant–stabilized S1 was obtained from The Native Antigen Company. Enzyme immunoassays were carried out as described previously (*42*). In brief, recombinant proteins were immobilized onto microtiter plate wells and used to capture spike-specific antibodies present in indicated dilutions of human serum samples. Captured IgG was detected using a human IgG–specific horseradish peroxidase–conjugated antibody followed by the addition of ultra-3,3′,5,5′-tetramethylbenzidine substrate (Thermo Fisher Scientific). Absorbance at 450 nm was read using a GlowMax Discover spectrophotometer (Promega). Data were presented as the ratio of the absorbance (OD_450_) for the sample versus the mean absorbance of three separate pools (*n* = 21) of negative control serum samples.

### Pseudovirus neutralization assay

Pseudovirus neutralization assays were performed as previously described (*43*). Briefly, 1.5 × 10^6^ HEK293T cells were seeded overnight in 8 ml of complete DMEM [Dulbecco’s modified Eagle’s medium supplemented with 10% fetal bovine serum and 1% nonessential amino acids] in a 10-cm-diameter Primaria-coated dish (Corning). Transfections were performed with 2 μg of murine leukemia virus Gag-Pol packaging vector (phCMV-5349), 2 μg of luciferase-encoding reporter plasmid (pTG126), and 2 μg of a plasmid encoding SARS-CoV-2 spike (lineage A, B.1.351, or P.1) with 24 μl of polyethylenimine (Polysciences) in Opti-MEM (Gibco). A no-envelope control (empty pseudotype) was used as a negative control in all experiments. Supernatants containing pseudotypes were harvested at 72 hours after transfection and filtered through 0.45-μm membranes. For neutralization assays, 2 × 10^4^ VeroE6 cells per well were plated in 100 μl of complete DMEM in white 96-well tissue culture plates (Corning) and incubated overnight at 37°C. The following day, supernatants containing SARS-CoV-2 pseudoviruses were incubated for 1 hour at room temperature with heat-inactivated serum samples at a 1:200 dilution or serially diluted before adding the samples to the VeroE6 cells. VeroE6 cells were incubated with inocula for 4 hours, which was then discarded and replaced with 200 μl of fresh complete DMEM. After 72 hours, the medium was discarded, cells were lysed with cell lysis buffer (Promega), and relative luciferase activity was measured using a FLUOstar Omega plate reader (BMG Labtech). Neutralizing activities were reported as reciprocal serum dilution corresponding to ID_50_ values and were calculated by nonlinear regression (GraphPad Prism version 9.1.2) using lower and upper bounds (0 and 100% inhibition) as constraints to assist curve fitting. Each sample was tested in triplicate.

### Statistical analysis

Data are presented as scatter plots with mean and SD, unless otherwise stated in the figure legends. Data were tested for normality and lognormality with D’Agostino and Pearson tests. Serology data were analyzed using Kruskal-Wallis with Dunn’s multiple comparisons test. ID_50_ data were log-transformed and analyzed with either one-way analysis of variance (ANOVA) with Tukey’s multiple comparisons test or two-way ANOVA with Tukey’s multiple comparisons test. Proportion neutralization data were analyzed using one-way ANOVA with Tukey’s multiple comparisons test. EC_50_ data were analyzed using Mann-Whitney two-tailed test. Data analysis was blinded. Correlation between antibody concentrations and neutralizing ID_50_ values was assessed using a Pearson correlation coefficient. Each experiment was performed once in either duplicate (serology) or triplicate (pseudovirus neutralization). Sample sizes were *n* = 25 for uninfected and *n* = 20 for infected, unless otherwise stated in the figure legends. Differences in means were considered statistically significant at *P* < 0.05. Significance levels are indicated as *P < 0.05; **P < 0.01; ***P < 0.001; ****P < 0.0001; ns, nonsignificant. Analyses were performed using GraphPad Prism 9.1.0 software.

## Notes

View/request a protocol for this paper from *Bio-protocol*.

## References

[1] E. E. Walsh, R. W. Frenck Jr., A. R. Falsey, N. Kitchin, J. Absalon, A. Gurtman, S. Lockhart, K. Neuzil, M. J. Mulligan, R. Bailey, K. A. Swanson, P. Li, K. Koury, W. Kalina, D. Cooper, C. Fontes-Garfias, P.-Y. Shi, Ö. Türeci, K. R. Tompkins, K. E. Lyke, V. Raabe, P. R. Dormitzer, K. U. Jansen, U. Şahin, W. C. Gruber, Safety and immunogenicity of two RNA-based Covid-19 vaccine candidates. N. Engl. J. Med. 383, 2439–2450 (2020). 33053279 10.1056/NEJMoa2027906 PMC7583697

[2] L. R. Baden, H. M. El Sahly, B. Essink, K. Kotloff, S. Frey, R. Novak, D. Diemert, S. A. Spector, N. Rouphael, C. B. Creech, J. McGettigan, S. Khetan, N. Segall, J. Solis, A. Brosz, C. Fierro, H. Schwartz, K. Neuzil, L. Corey, P. Gilbert, H. Janes, D. Follmann, M. Marovich, J. Mascola, L. Polakowski, J. Ledgerwood, B. S. Graham, H. Bennett, R. Pajon, C. Knightly, B. Leav, W. Deng, H. Zhou, S. Han, M. Ivarsson, J. Miller, T. Zaks; COVE Study Group, Efficacy and safety of the mRNA-1273 SARS-CoV-2 vaccine. N. Engl. J. Med. 384, 403–416 (2020). 33378609 10.1056/NEJMoa2035389 PMC7787219

[3] F. P. Polack, S. J. Thomas, N. Kitchin, J. Absalon, A. Gurtman, S. Lockhart, J. L. Perez, G. Pérez Marc, E. D. Moreira, C. Zerbini, R. Bailey, K. A. Swanson, S. Roychoudhury, K. Koury, P. Li, W. V. Kalina, D. Cooper, R. W. Frenck, L. L. Hammitt, Ö. Türeci, H. Nell, A. Schaefer, S. Ünal, D. B. Tresnan, S. Mather, P. R. Dormitzer, U. Şahin, K. U. Jansen, W. C. Gruber; C4591001 Clinical Trial Group, Safety and efficacy of the BNT162b2 mRNA Covid-19 vaccine. N. Engl. J. Med. 383, 2603–2615 (2020). 33301246 10.1056/NEJMoa2034577 PMC7745181

[4] M. N. Ramasamy, A. M. Minassian, K. J. Ewer, A. L. Flaxman, P. M. Folegatti, D. R. Owens, M. Voysey, P. K. Aley, B. Angus, G. Babbage, S. Belij-Rammerstorfer, L. Berry, S. Bibi, M. Bittaye, K. Cathie, H. Chappell, S. Charlton, P. Cicconi, E. A. Clutterbuck, R. Colin-Jones, C. Dold, K. R. W. Emary, S. Fedosyuk, M. Fuskova, D. Gbesemete, C. Green, B. Hallis, M. M. Hou, D. Jenkin, C. C. D. Joe, E. J. Kelly, S. Kerridge, A. M. Lawrie, A. Lelliott, M. N. Lwin, R. Makinson, N. G. Marchevsky, Y. Mujadidi, A. P. S. Munro, M. Pacurar, E. Plested, J. Rand, T. Rawlinson, S. Rhead, H. Robinson, A. J. Ritchie, A. L. Ross-Russell, S. Saich, N. Singh, C. C. Smith, M. D. Snape, R. Song, R. Tarrant, Y. Themistocleous, K. M. Thomas, T. L. Villafana, S. C. Warren, M. E. E. Watson, A. D. Douglas, A. V. S. Hill, T. Lambe, S. C. Gilbert, S. N. Faust, A. J. Pollard; Oxford COVID Vaccine Trail Group, Safety and immunogenicity of ChAdOx1 nCoV-19 vaccine administered in a prime-boost regimen in young and old adults (COV002): A single-blind, randomised, controlled, phase 2/3 trial. Lancet 396, 1979–1993 (2020). 33220855 10.1016/S0140-6736(20)32466-1 PMC7674972

[5] N. K. Jones, L. Rivett, S. Seaman, R. J. Samworth, B. Warne, C. Workman, M. Ferris, J. Wright, N. Quinnell, A. Shaw; Cambridge COVID-19 Collaboration, I. G. Goodfellow, P. J. Lehner, R. Howes, G. Wright, N. J. Matheson, M. P. Weekes, Single-dose BNT162b2 vaccine protects against asymptomatic SARS-CoV-2 infection. eLife 10, e68808 (2021). 33830018 10.7554/eLife.68808 PMC8064747

[6] P. M. Folegatti, K. J. Ewer, P. K. Aley, B. Angus, S. Becker, S. Belij-Rammerstorfer, D. Bellamy, S. Bibi, M. Bittaye, E. A. Clutterbuck, C. Dold, S. N. Faust, A. Finn, A. L. Flaxman, B. Hallis, P. Heath, D. Jenkin, R. Lazarus, R. Makinson, A. M. Minassian, K. M. Pollock, M. Ramasamy, H. Robinson, M. Snape, R. Tarrant, M. Voysey, C. Green, A. D. Douglas, A. V. S. Hill, T. Lambe, S. C. Gilbert, A. J. Pollard; Oxford COVID Vaccine Trial Group, Safety and immunogenicity of the ChAdOx1 nCoV-19 vaccine against SARS-CoV-2: A preliminary report of a phase 1/2, single-blind, randomised controlled trial. Lancet 396, 467–478 (2020). 32702298 10.1016/S0140-6736(20)31604-4 PMC7445431

[7] K. Wu, A. P. Werner, J. I. Moliva, M. Koch, A. Choi, G. B. E. Stewart-Jones, H. Bennett, S. Boyoglu-Barnum, W. Shi, B. S. Graham, A. Carfi, K. S. Corbett, R. A. Seder, D. K. Edwards, mRNA-1273 vaccine induces neutralizing antibodies against spike mutants from global SARS-CoV-2 variants. bioRxiv 2021.01.25.427948 [**Preprint**]. 25 January 2021. 10.1101/2021.01.25.427948.

[8] H. Tegally, E. Wilkinson, M. Giovanetti, A. Iranzadeh, V. Fonseca, J. Giandhari, D. Doolabh, S. Pillay, E. J. San, N. Msomi, K. Mlisana, A. von Gottberg, S. Walaza, M. Allam, A. Ismail, T. Mohale, A. J. Glass, S. Engelbrecht, G. Van Zyl, W. Preiser, F. Petruccione, A. Sigal, D. Hardie, G. Marais, N.-y. Hsiao, S. Korsman, M.-A. Davies, L. Tyers, I. Mudau, D. York, C. Maslo, D. Goedhals, S. Abrahams, O. Laguda-Akingba, A. Alisoltani-Dehkordi, A. Godzik, C. K. Wibmer, B. T. Sewell, J. Lourenço, L. C. J. Alcantara, S. L. K. Pond, S. Weaver, D. Martin, R. J. Lessells, J. N. Bhiman, C. Williamson, T. de Oliveira, Detection of a SARS-CoV-2 variant of concern in South Africa. Nature 592, 438–443 (2021). 33690265 10.1038/s41586-021-03402-9

[9] Y. Weisblum, F. Schmidt, F. Zhang, J. DaSilva, D. Poston, J. C. C. Lorenzi, F. Muecksch, M. Rutkowska, H.-H. Hoffmann, E. Michailidis, C. Gaebler, M. Agudelo, A. Cho, Z. Wang, A. Gazumyan, M. Cipolla, L. Luchsinger, C. D. Hillyer, M. Caskey, D. F. Robbiani, C. M. Rice, M. C. Nussenzweig, T. Hatziioannou, P. D. Bieniasz, Escape from neutralizing antibodies by SARS-CoV-2 spike protein variants. eLife 9, e61312 (2020). 33112236 10.7554/eLife.61312 PMC7723407

[10] C. K. Wibmer, F. Ayres, T. Hermanus, M. Madzivhandila, P. Kgagudi, B. Oosthuysen, B. E. Lambson, T. de Oliveira, M. Vermeulen, K. van der Berg, T. Rossouw, M. Boswell, V. Ueckermann, S. Meiring, A. von Gottberg, C. Cohen, L. Morris, J. N. Bhiman, P. L. Moore, SARS-CoV-2 501Y.V2 escapes neutralization by South African COVID-19 donor plasma. Nat. Med. 27, 622–625 (2021). 33654292 10.1038/s41591-021-01285-x

[11] M. Voysey, S. A. C. Clemens, S. A. Madhi, L. Y. Weckx, P. M. Folegatti, P. K. Aley, B. Angus, V. L. Baillie, S. L. Barnabas, Q. E. Bhorat, S. Bibi, C. Briner, P. Cicconi, E. A. Clutterbuck, A. M. Collins, C. L. Cutland, T. C. Darton, K. Dheda, C. Dold, C. J. A. Duncan, K. R. W. Emary, K. J. Ewer, A. Flaxman, L. Fairlie, S. N. Faust, S. Feng, D. M. Ferreira, A. Finn, E. Galiza, A. L. Goodman, C. M. Green, C. A. Green, M. Greenland, C. Hill, H. C. Hill, I. Hirsch, A. Izu, D. Jenkin, C. C. D. Joe, S. Kerridge, A. Koen, G. Kwatra, R. Lazarus, V. Libri, P. J. Lillie, N. G. Marchevsky, R. P. Marshall, A. V. A. Mendes, E. P. Milan, A. M. Minassian, A. McGregor, Y. F. Mujadidi, A. Nana, S. D. Padayachee, D. J. Phillips, A. Pittella, E. Plested, K. M. Pollock, M. N. Ramasamy, A. J. Ritchie, H. Robinson, A. V. Schwarzbold, A. Smith, R. Song, M. D. Snape, E. Sprinz, R. K. Sutherland, E. C. Thomson, M. E. Török, M. Toshner, D. P. J. Turner, J. Vekemans, T. L. Villafana, T. White, C. J. Williams, A. D. Douglas, A. V. S. Hill, T. Lambe, S. C. Gilbert, A. J. Pollard; Oxford COVID Vaccine Trail Group, Single-dose administration and the influence of the timing of the booster dose on immunogenicity and efficacy of ChAdOx1 nCoV-19 (AZD1222) vaccine: A pooled analysis of four randomised trials. Lancet 397, 881–891 (2021). 33617777 10.1016/S0140-6736(21)00432-3 PMC7894131

[12] W. F. Garcia-Beltran, E. C. Lam, K. S. Denis, A. D. Nitido, Z. H. Garcia, B. M. Hauser, J. Feldman, M. N. Pavlovic, D. J. Gregory, M. C. Poznansky, A. Sigal, A. G. Schmidt, A. J. Iafrate, V. Naranbhai, A. B. Balazs, Multiple SARS-CoV-2 variants escape neutralization by vaccine-induced humoral immunity. Cell 184, 2372–2383.e9 (2021). 33743213 10.1016/j.cell.2021.03.013 PMC7953441

[13] T. Kustin, N. Harel, U. Finkel, S. Perchik, S. Harari, M. Tahor, I. Caspi, R. Levy, M. Leschinsky, S. K. Dror, G. Bergerzon, H. Gadban, F. Gadban, E. Eliassian, O. Shimron, L. Saleh, H. Ben-Zvi, D. Amichay, A. Ben-Dor, D. Sagas, M. Strauss, Y. S. Avni, A. Huppert, E. Kepten, R. D. Balicer, D. Nezer, S. Ben-Shachar, A. Stern, Evidence for increased breakthrough rates of SARS-CoV-2 variants of concern in BNT162b2-mRNA-vaccinated individuals. Nat. Med. 27, 1379–1384 (2021). 34127854 10.1038/s41591-021-01413-7 PMC8363499

[14] K. Wu, A. Choi, M. Koch, S. Elbashir, L. Ma, D. Lee, A. Woods, C. Henry, C. Palandjian, A. Hill, J. Quinones, N. Nunna, S. O’Connell, A. B. McDermott, S. Falcone, E. Narayanan, T. Colpitts, H. Bennett, K. S. Corbett, R. Seder, B. S. Graham, G. B. Stewart-Jones, A. Carfi, D. K. Edwards, Variant SARS-CoV-2 mRNA vaccines confer broad neutralization as primary or booster series in mice. bioRxiv 2021.04.13.439482 [**Preprint**]. 13 April 2021. 10.1101/2021.04.13.439482.

[15] S. Ranjeva, R. Subramanian, V. J. Fang, G. M. Leung, D. K. M. Ip, R. A. P. M. Perera, J. S. M. Peiris, B. J. Cowling, S. Cobey, Age-specific differences in the dynamics of protective immunity to influenza. Nat. Commun. 10, 1660 (2019). 30971703 10.1038/s41467-019-09652-6 PMC6458119

[16] A. M. Valdes, J. C. Moon, A. Vijay, N. Chaturvedi, A. Norrish, A. Ikram, S. Craxford, L. M. L. Cusin, J. Nightingale, A. Semper, T. Brooks, A. McKnight, H. Kurdi, C. Menni, P. Tighe, M. Noursadeghi, G. Aithal, T. A. Treibel, B. J. Ollivere, C. Manisty, Longitudinal assessment of symptoms and risk of SARS-CoV-2 infection in healthcare workers across 5 hospitals to understand ethnic differences in infection risk. EClinicalMedicine 34, 100835 (2021). 33880438 10.1016/j.eclinm.2021.100835 PMC8049191

[17] S. Craxford, J. Nightingale, B. Marson, A. Vijay, A. Norrish, A. Ikram, L. M. L. Cusin, P. Tighe, G. P. Aithal, S. Astbury, J. K. Ball, J. Newham, R. A. Urbanowicz, A. Kelly, W. Ashraf, A. W. Tarr, A. M. Valdes, B. J. Ollivere, SARS-CoV-2 transmission from the healthcare setting into the home: A prospective longitudinal cohort study. medRxiv 10.1101/2021.02.01.21250950 (2021).

[18] Y. Kaku, T. Kuwata, H. M. Zahid, T. Hashiguchi, T. Noda, N. Kuramoto, S. Biswas, K. Matsumoto, M. Shimizu, Y. Kawanami, K. Shimura, C. Onishi, Y. Muramoto, T. Suzuki, J. Sasaki, Y. Nagasaki, R. Minami, C. Motozono, M. Toyoda, H. Takahashi, H. Kishi, K. Fujii, T. Tatsuke, T. Ikeda, Y. Maeda, T. Ueno, Y. Koyanagi, H. Iwagoe, S. Matsushita, Resistance of SARS-CoV-2 variants to neutralization by antibodies induced in convalescent patients with COVID-19. Cell Rep. 36, 109385 (2021). 34237284 10.1016/j.celrep.2021.109385 PMC8226103

[19] A. B. Vogel, I. Kanevsky, Y. Che, K. A. Swanson, A. Muik, M. Vormehr, L. M. Kranz, K. C. Walzer, S. Hein, A. Güler, J. Loschko, M. S. Maddur, A. Ota-Setlik, K. Tompkins, J. Cole, B. G. Lui, T. Ziegenhals, A. Plaschke, D. Eisel, S. C. Dany, S. Fesser, S. Erbar, F. Bates, D. Schneider, B. Jesionek, B. Sänger, A.-K. Wallisch, Y. Feuchter, H. Junginger, S. A. Krumm, A. P. Heinen, P. Adams-Quack, J. Schlereth, S. Schille, C. Kröner, R. de la Caridad Güimil Garcia, T. Hiller, L. Fischer, R. S. Sellers, S. Choudhary, O. Gonzalez, F. Vascotto, M. R. Gutman, J. A. Fontenot, S. Hall-Ursone, K. Brasky, M. C. Griffor, S. Han, A. A. H. Su, J. A. Lees, N. L. Nedoma, E. H. Mashalidis, P. V. Sahasrabudhe, C. Y. Tan, D. Pavliakova, G. Singh, C. Fontes-Garfias, M. Pride, I. L. Scully, T. Ciolino, J. Obregon, M. Gazi, R. Carrion, K. J. Alfson, W. V. Kalina, D. Kaushal, P.-Y. Shi, T. Klamp, C. Rosenbaum, A. N. Kuhn, Ö. Türeci, P. R. Dormitzer, K. U. Jansen, U. Sahin, BNT162b vaccines protect rhesus macaques from SARS-CoV-2. Nature 592, 283–289 (2021). 33524990 10.1038/s41586-021-03275-y

[20] M. K. Slifka, R. Antia, J. K. Whitmire, R. Ahmed, Humoral immunity due to long-lived plasma cells. Immunity 8, 363–372 (1998). 9529153 10.1016/s1074-7613(00)80541-5

[21] M. Prendecki, C. Clarke, J. Brown, A. Cox, S. Gleeson, M. Guckian, P. Randell, A. D. Pria, L. Lightstone, X.-N. Xu, W. Barclay, S. P. McAdoo, P. Kelleher, M. Willicombe, Effect of previous SARS-CoV-2 infection on humoral and T-cell responses to single-dose BNT162b2 vaccine. Lancet 397, 1178–1181 (2021). 33640037 10.1016/S0140-6736(21)00502-X PMC7993933

[22] U. Sahin, A. Muik, I. Vogler, E. Derhovanessian, L. M. Kranz, M. Vormehr, J. Quandt, N. Bidmon, A. Ulges, A. Baum, K. E. Pascal, D. Maurus, S. Brachtendorf, V. Lörks, J. Sikorski, P. Koch, R. Hilker, D. Becker, A.-K. Eller, J. Grützner, M. Tonigold, C. Boesler, C. Rosenbaum, L. Heesen, M.-C. Kühnle, A. Poran, J. Z. Dong, U. Luxemburger, A. Kemmer-Brück, D. Langer, M. Bexon, S. Bolte, T. Palanche, A. Schultz, S. Baumann, A. J. Mahiny, G. Boros, J. Reinholz, G. T. Szabó, K. Karikó, P.-Y. Shi, C. Fontes-Garfias, J. L. Perez, M. Cutler, D. Cooper, C. A. Kyratsous, P. R. Dormitzer, K. U. Jansen, Ö. Türeci, BNT162b2 vaccine induces neutralizing antibodies and poly-specific T cells in humans. Nature 595, 572–577 (2021). 34044428 10.1038/s41586-021-03653-6

[23] C. J. Reynolds, C. Pade, J. M. Gibbons, D. K. Butler, A. D. Otter, K. Menacho, M. Fontana, A. Smit, J. E. Sackville-West, T. Cutino-Moguel, M. K. Maini, B. Chain, M. Noursadeghi, T. Brooks, A. Semper, C. Manisty, T. A. Treibel, J. C. Moon; UK COVIDsortium Investigators, A. M. Valdes, Á. McKnight, D. M. Altmann, R. Boyton, Prior SARS-CoV-2 infection rescues B and T cell responses to variants after first vaccine dose. Science 372, 1418–1423 (2021). 33931567 10.1126/science.abh1282 PMC8168614

[24] S. Cele, I. Gazy, L. Jackson, S. H. Hwa, H. Tegally, G. Lustig, J. Giandhari, S. Pillay, E. Wilkinson, Y. Naidoo, F. Karim, Y. Ganga, K. Khan, M. Bernstein, A. B. Balazs, B. I. Gosnell, W. Hanekom, M. S. Moosa; Network for Genomic Surveillance in South Africa; COMMIT-KZN Team, R. J. Lessells, T. de Oliveira, A. Sigal, Escape of SARS-CoV-2 501Y.V2 from neutralization by convalescent plasma. Nature 593, 142–146 (2021). 33780970 10.1038/s41586-021-03471-w PMC9867906

[25] L. Bian, F. Gao, J. Zhang, Q. He, Q. Mao, M. Xu, Z. Liang, Effects of SARS-CoV-2 variants on vaccine efficacy and response strategies. Expert Rev. Vaccines 20, 365–373 (2021). 33851875 10.1080/14760584.2021.1903879 PMC8054487

[26] T. Bradley, E. Grundberg, R. Selvarangan, C. LeMaster, E. Fraley, D. Banerjee, B. Belden, D. Louiselle, N. Nolte, R. Biswell, T. Pastinen, A. Myers, J. Schuster, Antibody responses after a single dose of SARS-CoV-2 mRNA vaccine. N. Engl. J. Med. 384, 1959–1961 (2021). 33755375 10.1056/NEJMc2102051 PMC8008753

[27] I. Hyseni, E. Molesti, L. Benincasa, P. Piu, E. Casa, N. J. Temperton, A. Manenti, E. Montomoli, Characterisation of SARS-CoV-2 lentiviral pseudotypes and correlation between pseudotype-based neutralisation assays and live virus-based micro neutralisation assays. Viruses 12, 1011 (2020). 32927639 10.3390/v12091011 PMC7551040

[28] L. du Plessis, J. T. McCrone, A. E. Zarebski, V. Hill, C. Ruis, B. Gutierrez, J. Raghwani, J. Ashworth, R. Colquhoun, T. R. Connor, N. R. Faria, B. Jackson, N. J. Loman, Á. O’Toole, S. M. Nicholls, K. V. Parag, E. Scher, T. I. Vasylyeva, E. M. Volz, A. Watts, I. I. Bogoch, K. Khan; COVID-19 Genomics UK (COG-UK) Consortium, D. M. Aanensen, M. U. G. Kraemer, A. Rambaut, O. G. Pybus, Establishment and lineage dynamics of the SARS-CoV-2 epidemic in the UK. Science 371, 708–712 (2021). 33419936 10.1126/science.abf2946 PMC7877493

[29] J. G. Chappell, T. Tsoleridis, G. Clark, L. Berry, N. Holmes, C. Moore, M. Carlile, F. Sang, B. J. Debebe, V. Wright, W. L. Irving, B. J. Thomson, T. C. J. Boswell, I. Willingham, A. Joseph, W. Smith, M. Khakh, V. M. Fleming, M. M. Lister, H. C. Howson-Wells, E. C. Holmes, M. W. Loose, J. K. Ball, C. P. McClure; On Behalf Of The Cog-Uk Consortium, Retrospective screening of routine respiratory samples revealed undetected community transmission and missed intervention opportunities for SARS-CoV-2 in the United Kingdom. J. Gen. Virol. 102, 10.1099/jgv.0.001595, (2021). 10.1099/jgv.0.001595 PMC8459093 34130773

[30] L. J. Abu-Raddad, H. Chemaitelly, A. A. Butt; National Study Group for COVID-19 Vaccination, Effectiveness of the BNT162b2 Covid-19 vaccine against the B.1.1.7 and B.1.351 variants. N. Engl. J. Med. 385, 187–189 (2021). 33951357 10.1056/NEJMc2104974 PMC8117967

[31] X. Shen, H. Tang, C. McDanal, K. Wagh, W. Fischer, J. Theiler, H. Yoon, D. Li, B. F. Haynes, K. O. Sanders, S. Gnanakaran, N. Hengartner, R. Pajon, G. Smith, G. M. Glenn, B. Korber, D. C. Montefiori, SARS-CoV-2 variant B.1.1.7 is susceptible to neutralizing antibodies elicited by ancestral spike vaccines. Cell Host Microbe 29, 529–539.e3 (2021). 33705729 10.1016/j.chom.2021.03.002 PMC7934674

[32] H. Parry, R. Bruton, C. Stephens, K. Brown, G. Amirthalingam, B. Hallis, A. Otter, J. Zuo, P. Moss, Extended interval BNT162b2 vaccination enhances peak antibody generation in older people. medRxiv 10.1101/2021.05.15.21257017 (2021). 10.1038/s41541-022-00432-w PMC8795435 35087066

[33] E. J. Anderson, N. G. Rouphael, A. T. Widge, L. A. Jackson, P. C. Roberts, M. Makhene, J. D. Chappell, M. R. Denison, L. J. Stevens, A. J. Pruijssers, A. B. McDermott, B. Flach, B. C. Lin, N. A. Doria-Rose, S. O’Dell, S. D. Schmidt, K. S. Corbett, P. A. Swanson, M. Padilla, K. M. Neuzil, H. Bennett, B. Leav, M. Makowski, J. Albert, K. Cross, V. V. Edara, K. Floyd, M. S. Suthar, D. R. Martinez, R. Baric, W. Buchanan, C. J. Luke, V. K. Phadke, C. A. Rostad, J. E. Ledgerwood, B. S. Graham, J. H. Beigel; mRNA-1273 Study Group, Safety and immunogenicity of SARS-CoV-2 mRNA-1273 vaccine in older adults. N. Engl. J. Med. 383, 2427–2438 (2020). 32991794 10.1056/NEJMoa2028436 PMC7556339

[34] D. Y. Logunov, I. V. Dolzhikova, D. V. Shcheblyakov, A. I. Tukhvatulin, O. V. Zubkova, A. S. Dzharullaeva, A. V. Kovyrshina, N. L. Lubenets, D. M. Grousova, A. S. Erokhova, A. G. Botikov, F. M. Izhaeva, O. Popova, T. A. Ozharovskaya, I. B. Esmagambetov, I. A. Favorskaya, D. I. Zrelkin, D. V. Voronina, D. N. Shcherbinin, A. S. Semikhin, Y. V. Simakova, E. A. Tokarskaya, D. A. Egorova, M. M. Shmarov, N. A. Nikitenko, V. A. Gushchin, E. A. Smolyarchuk, S. K. Zyryanov, S. V. Borisevich, B. S. Naroditsky, A. L. Gintsburg; Gam-COVID-Vac Vaccine Trial Group, Safety and efficacy of an rAd26 and rAd5 vector-based heterologous prime-boost COVID-19 vaccine: An interim analysis of a randomised controlled phase 3 trial in Russia. Lancet 397, 671–681 (2021). 33545094 10.1016/S0140-6736(21)00234-8 PMC7852454

[35] L. Stamatatos, J. Czartoski, Y.-H. Wan, L. J. Homad, V. Rubin, H. Glantz, M. Neradilek, E. Seydoux, M. F. Jennewein, A. J. MacCamy, J. Feng, G. Mize, S. C. De Rosa, A. Finzi, M. P. Lemos, K. W. Cohen, Z. Moodie, M. J. McElrath, A. T. McGuire, mRNA vaccination boosts cross-variant neutralizing antibodies elicited by SARS-CoV-2 infection. Science 372, eabg9175 (2021). 33766944 10.1126/science.abg9175 PMC8139425

[36] M. Velasco, M. I. Galán, M. L. Casas, E. Perez-Fernandez, D. Martínez-Ponce, B. González-Piñeiro, V. Castilla, C. Guijarro; Alcorcón COVID-19 Working Group, Impact of previous coronavirus disease 2019 on immune response after a single dose of BNT162b2 severe acute respiratory syndrome coronavirus 2 vaccine. Open Forum Infect. Dis. 8, ofab299 (2021). 34258322 10.1093/ofid/ofab299 PMC8244747

[37] A. R. Demonbreun, A. Sancilio, M. P. Velez, D. T. Ryan, R. Saber, L. A. Vaught, N. L. Reiser, R. R. Hsieh, R. T. D'Aquila, B. Mustanski, E. M. McNally, T. W. McDade, Comparison of IgG and neutralizing antibody responses after one or two doses of COVID-19 mRNA vaccine in previously infected and uninfected individuals. EClinicalMedicine 38, 101018 (2021). 34278286 10.1016/j.eclinm.2021.101018 PMC8276631

[38] F. Gobbi, D. Buonfrate, L. Moro, P. Rodari, C. Piubelli, S. Caldrer, S. Riccetti, A. Sinigaglia, L. Barzon, Antibody response to the BNT162b2 mRNA COVID-19 vaccine in subjects with prior SARS-CoV-2 infection. Viruses 13, (2021). 10.3390/v13030422 PMC8001674 33807957

[39] A. Angyal, S. Longet, S. Moore, R. Payne, A. Harding, T. Tipton, P. Rongkard, M. Ali, L. Hering, N. Meardon, J. Austin, R. Brown, D. Skelly, N. Gillson, S. Dobson, A. Cross, G. Sandhar, J. Kilby, J. Tyerman, A. Nicols, J. Spegarova, H. Mehta, H. Hornsby, R. Whitham, C. Conlon, K. Jeffery, P. Goulder, J. Frater, C. Dold, M. Pace, A. Ogbe, H. Brown, A. Ansari, E. Adland, A. Brown, M. Chand, A. Shields, P. Matthews, S. Hopkins, V. J. Hall, W. James, S. Rowland-Jones, P. Klenerman, S. Dunachie, A. Richter, C. Duncan, E. Barnes, M. Carroll, L. Turtle, T. d. Silva, PITCH Consortium Group. T-cell and antibody responses to first BNT162b2 vaccine dose in previously SARS-CoV-2-infected and infection-naive UK healthcare workers: a multicentre, prospective, observational cohort study. SSRN. 2021; (published online April 13.) (preprint). https://papers.ssrn.com/sol3/papers.cfm?abstract_id=3820576.

[40] J. M. Dan, J. Mateus, Y. Kato, K. M. Hastie, E. D. Yu, C. E. Faliti, A. Grifoni, S. I. Ramirez, S. Haupt, A. Frazier, C. Nakao, V. Rayaprolu, S. A. Rawlings, B. Peters, F. Krammer, V. Simon, E. O. Saphire, D. M. Smith, D. Weiskopf, A. Sette, S. Crotty, Immunological memory to SARS-CoV-2 assessed for up to 8 months after infection. Science 371, eabf4063 (2021). 33408181 10.1126/science.abf4063 PMC7919858

[41] C. Manisty, A. D. Otter, T. A. Treibel, Á. McKnight, D. M. Altmann, T. Brooks, M. Noursadeghi, R. J. Boyton, A. Semper, J. C. Moon, Antibody response to first BNT162b2 dose in previously SARS-CoV-2-infected individuals. Lancet 397, 1057–1058 (2021). 33640038 10.1016/S0140-6736(21)00501-8 PMC7972310

[42] P. J. Tighe, R. A. Urbanowicz, C. L. Fairclough, C. P. McClure, B. J. Thomson, N. Gomez, J. G. Chappell, T. Tsoleridis, M. Loose, M. Carlile, C. Moore, N. Holmes, F. Sang, D. Hrushikesh, G. Clark, N. Temperton, T. Brooks, J. K. Ball, W. L. Irving, A. W. Tarr, Potent anti-SARS-CoV-2 antibody responses are associated with better prognosis in hospital inpatient COVID-19 disease. medRxiv 10.1101/2020.08.22.20176834 (2020).

[43] S. Capone, A. Raggioli, M. Gentile, S. Battella, A. Lahm, A. Sommella, A. M. Contino, R. A. Urbanowicz, R. Scala, F. Barra, A. Leuzzi, E. Lilli, G. Miselli, A. Noto, M. Ferraiuolo, F. Talotta, T. Tsoleridis, C. Castilletti, G. Matusali, F. Colavita, D. Lapa, S. Meschi, M. Capobianchi, M. Soriani, A. Folgori, J. K. Ball, S. Colloca, A. Vitelli, Immunogenicity of a new gorilla adenovirus vaccine candidate for COVID-19. Mol. Ther. 29, 2412–2423 (2021). 33895322 10.1016/j.ymthe.2021.04.022 PMC8062434

