# Supplementary material for: Two doses of the SARS-CoV-2 BNT162b2 vaccine enhance antibody responses to variants in individuals with prior SARS-CoV-2 infection
Source: Sci Transl Med. 2021 Aug 10;13(609):eabj0847. doi: 10.1126/scitranslmed.abj0847 (PMC9835846; doi:10.1126/scitranslmed.abj0847)
Supplement: Supplementary file 1 — Figs. S1 to S5 Tables S1 and S2 [file scitranslmed.abj0847_sm.pdf]

Science Translational Medicine
AAAS

Supplementary Materials for

Two doses of the SARS-CoV-2 BNT162b2 vaccine enhance antibody responses to variants in individuals with prior SARS-CoV-2 infection

Richard A. Urbanowicz *et al*.

Corresponding author: Jonathan K. Ball, jonathan.ball@nottingham.ac.uk; Ana M. Valdes, ana.valdes@nottingham.ac.uk; Benjamin J. Ollivere, benjamin.ollivere@nottingham.ac.uk

Sci. Transl. Med. **13**, eabj0847 (2021)
DOI: 10.1126/scitranslmed.abj0847

The PDF file includes:

Figs. S1 to S5
Tables S1 and S2

Other Supplementary Material for this manuscript includes the following:

Data file S1

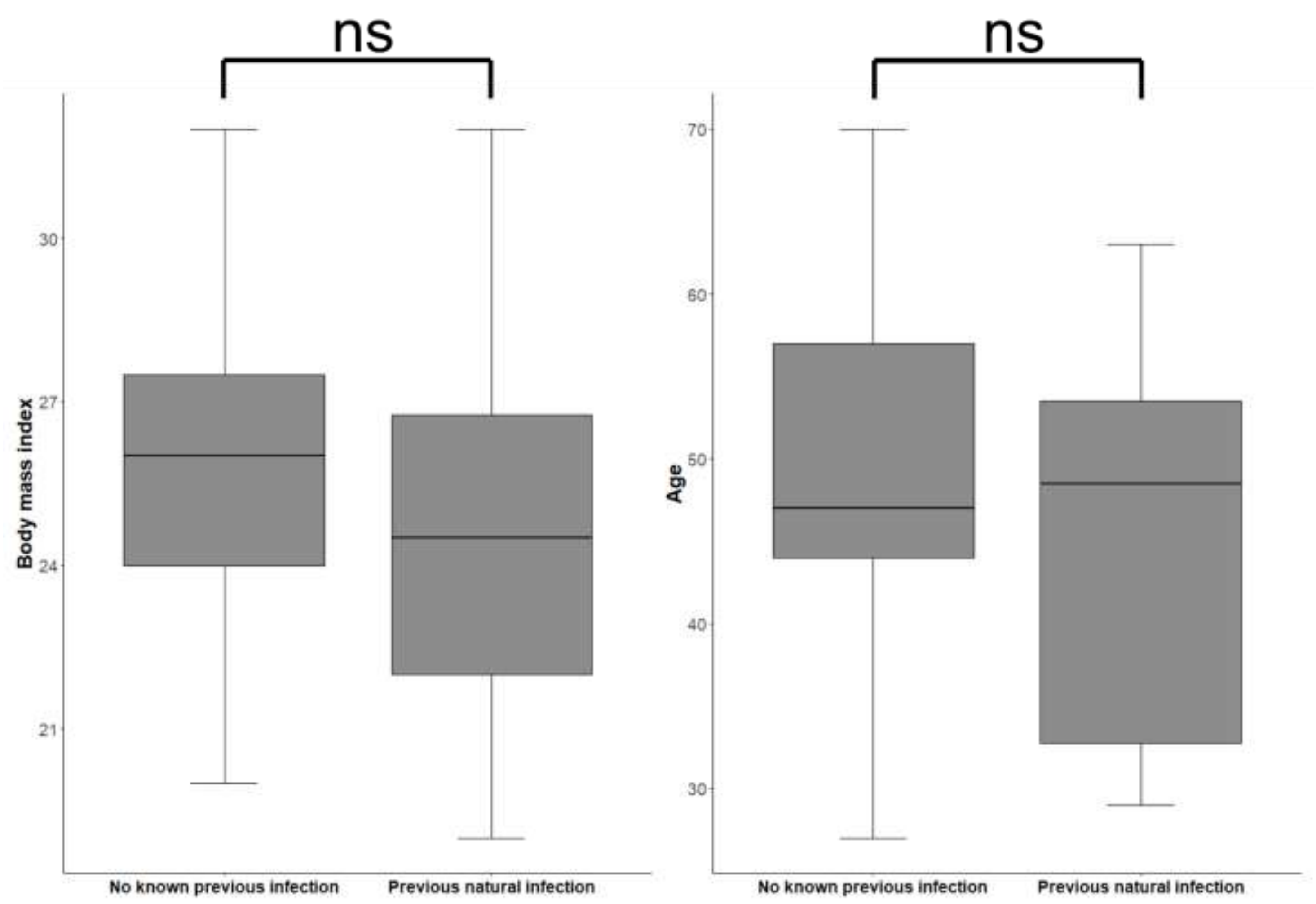

Fig. S1. Body mass index and age difference in cohort individuals who had no previous SARS-CoV-2 infection versus those with a previous infection. Significance was evaluated using a t-test; ns, not significant.

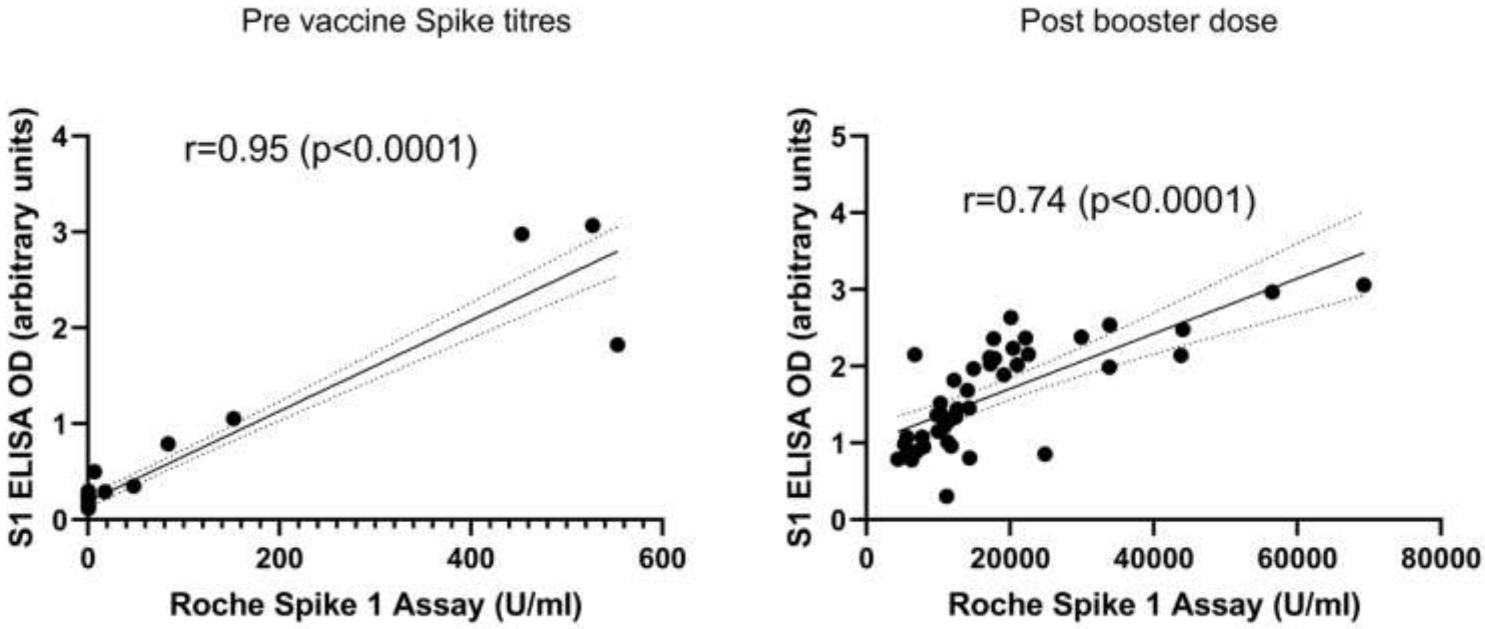

Fig. S2. Validation of in-house S1 assay versus a commercially available spike antibody assay (Roche). Equivalent serum samples obtained either pre-vaccination (**A**) or post-boost vaccination (**B**) were run in parallel on either the in-house or Roche antibody assays. Parametric linear regression (r value) and p values are shown. ELISA, enzyme-linked immunosorbent assay; OD, optical density; U, units.

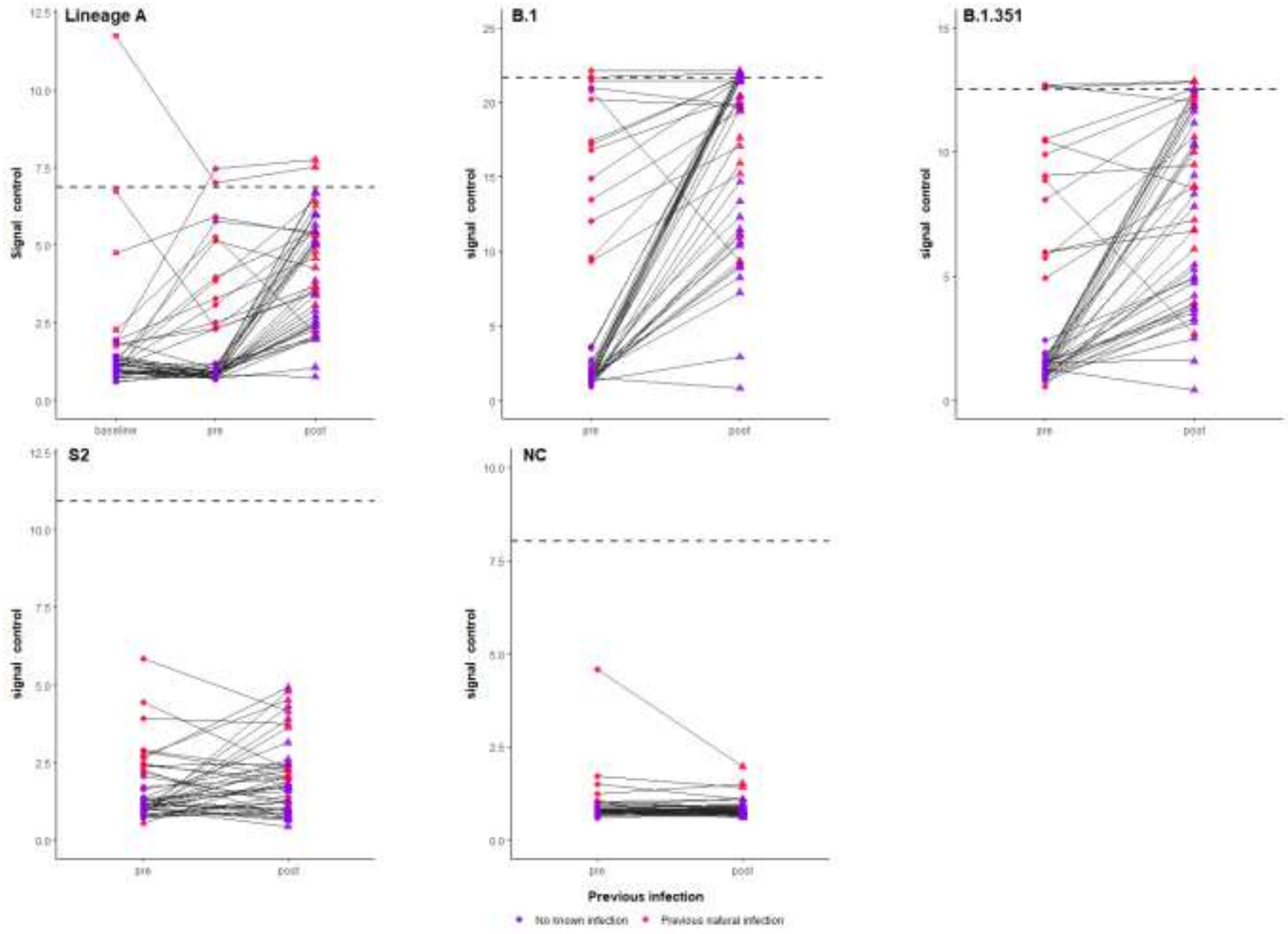

Fig. S3. Antibody reactivity to different SARS-CoV2 S1 variants and to other viral proteins. The ratio of reactivity to S1, S2, and nucleocapsid (NC) to control at 1:600 dilution of was measured by ELISA using serum samples at indicated time points before the first dose of vaccine (baseline), after the first dose but two days before the second dose (pre) or 14 days after the second dose (post). Infection state is denoted by color. Due to sample constraints, n=24 for pre with no known infection. The dotted line represents a positive control sample.

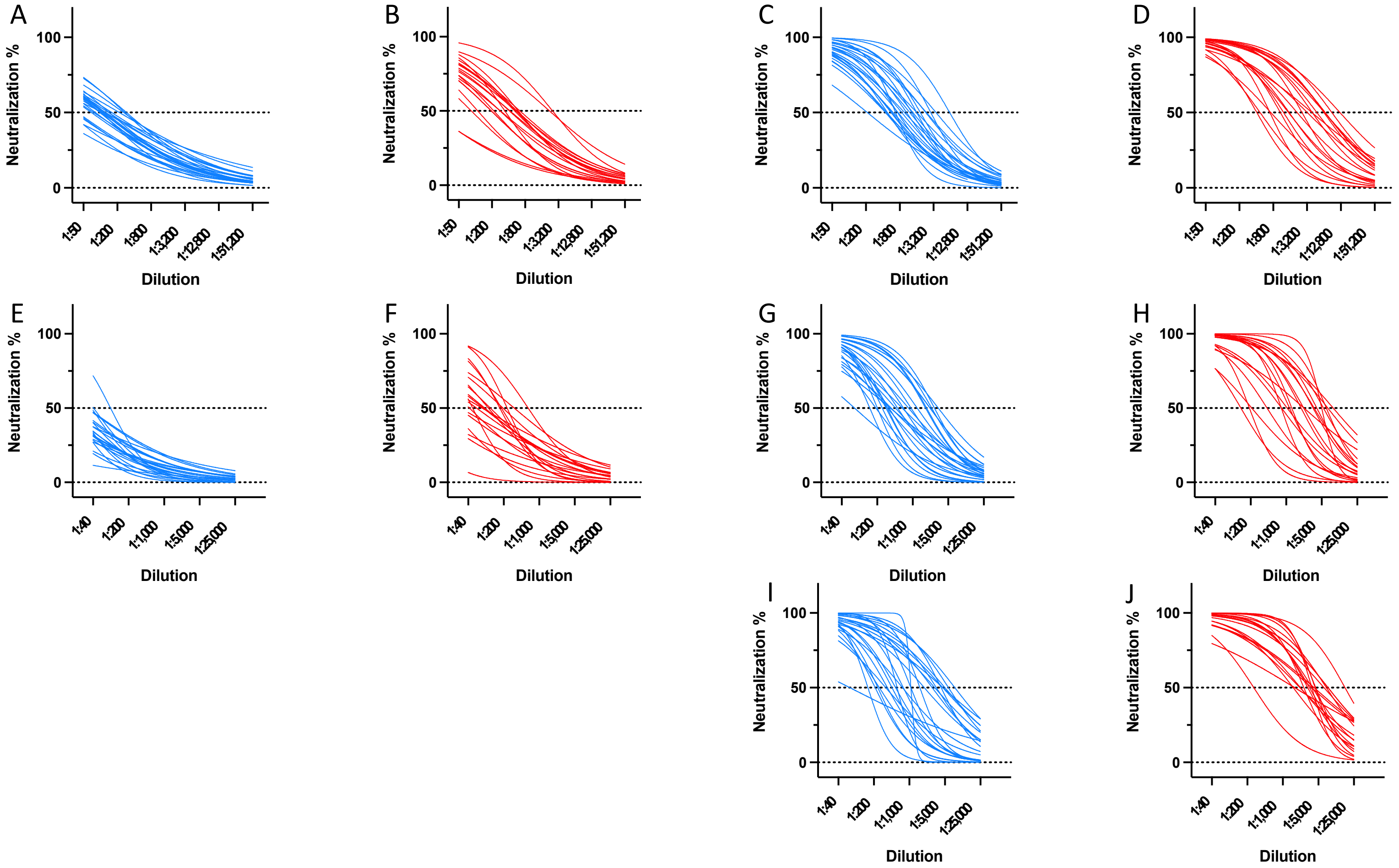

Fig. S4. Nonlinear regression curves used ot generate ID_{50} values for Fig. 3. (A to D) Neutralization of Lineage A by serum samples collected after one dose of vaccine without infection (A) or with infection (B) and after two doses of vaccine without infection (C) or with infection (D). **(E to H)** Neutralization of B.1.351 by serum samples collected after one dose of vaccine without infection (E) or with infection (F) and after two doses of vaccine without infection (G) or with infection (H). **(I and J)** Neutralization of P.1 by serum samples collected after two doses of vaccine without infection (I) or with infeciton (J). Dotted lines represent the concentration of serum required to inhibit infection by 50% (ID_{50}).

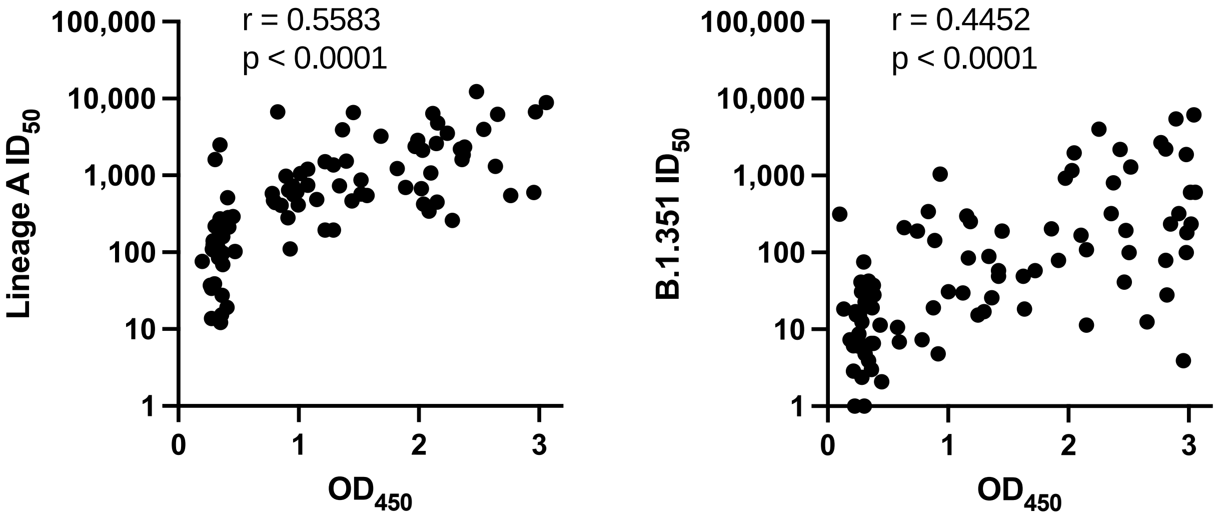

Fig. S5. Correlation plots of reactivity to S1 and ID_{50}. (**A and B**) Correlation between reactivity to S1 as measured by OD_{450} and ID_{50} is shown for Lineage A (A) and B.1.351 (B). Pearson's correlation coefficient (r value) and p values are shown.

Table S1. Cohort characteristics. PCR, polymerase chain reaction.

	History of SARS-CoV-2 infection (n=20)	No history of infection (n=25)
Age	48.5 (29 - 63)	47 (27 - 70)
Gender (M/F)	3/17	6/19
Body Mass Index	25.1 ± 3.76	26.0 ± 3.21
Timing of baseline sampling (# days before 1st vaccine dose)	219 (195-239)	219 (209-239)
Timing of pre-boost sampling (# days after 1st vaccine dose)	68 (58 - 72)	68 (49 - 71)
Timing of post-boost sampling (# days after 2nd vaccine dose)	18 (13 - 29)	19 (14 - 25)
# days between vaccine doses	70 (67 - 75)	70 (68 - 73)
Diagnosis (PCR + serology; PCR only; Serology only)	6; 3; 11	N/A
First diagnosed		
Q1 (January to March 2020)	0	
Q2 (April to June 2020)	12	
Q3 (July to September 2020)	4	
Q4 (October to December 2020)	4	
Ethnicity		
White/Caucasian	19	22
Asian/other	0	2
Indian	1	1

**Table S2. Statistical comparisons for single dilution point neutralization assays presented in Fig. 2C. ***p<0.05, **p<0.01, ***p<0.005, ****p<0.0001, ns, not significant.

**Tukey's multiple comparisons test**	**Summary**	**Adjusted P Value**
Lineage A Prior SARS-CoV-2, One Vaccine Dose versus Lineage A Naïve, One Vaccine Dose	ns	>0.9999
Lineage A Prior SARS-CoV-2, One Vaccine Dose versus B.1.351 Prior SARS-CoV-2, One Vaccine Dose	**	0.001
Lineage A Prior SARS-CoV-2, One Vaccine Dose versus B.1.351 Naïve, One Vaccine One Dose	ns	>0.9999
Lineage A Prior SARS-CoV-2, One Vaccine Dose versus P.1 Prior SARS-CoV-2, One Vaccine Dose	ns	>0.9999
Lineage A Prior SARS-CoV-2, One Vaccine Dose versus P.1 Naïve, One Vaccine Dose	ns	>0.9999
Lineage A Prior SARS-CoV-2, One Vaccine Dose versus Lineage A Prior SARS-CoV-2, Two Vaccine Doses	****	<0.0001
Lineage A Prior SARS-CoV-2, One Vaccine Dose versus Lineage A Naïve, Two Vaccine Doses	ns	>0.9999
Lineage A Prior SARS-CoV-2, One Vaccine Dose versus B.1.351 Prior SARS-CoV-2, Two Vaccine Doses	****	<0.0001
Lineage A Prior SARS-CoV-2, One Vaccine Dose versus B.1.351 Naïve, Two Vaccine Doses	ns	>0.9999
Lineage A Prior SARS-CoV-2, One Vaccine Dose versus P.1 Prior SARS-CoV-2, Two Vaccine Doses	****	<0.0001
Lineage A Prior SARS-CoV-2, One Vaccine Dose versus P.1 Naïve, Two Vaccine Doses	ns	>0.9999
Lineage A Naïve, One Vaccine Dose versus B.1.351 Prior SARS-CoV-2, One Vaccine Dose	ns	>0.9999
Lineage A Naïve, One Vaccine Dose versus B.1.351 Naïve, One Vaccine Dose	****	<0.0001
Lineage A Naïve, One Vaccine Dose versus P.1 Prior SARS-CoV-2, One Vaccine Dose	ns	>0.9999
Lineage A Naïve, One Vaccine Dose versus P.1 Naïve, One Vaccine Dose	ns	>0.9999
Lineage A Naïve, One Vaccine Dose versus Lineage A Prior SARS-CoV-2, Two Vaccine Doses	ns	>0.9999
Lineage A Naïve, One Vaccine Dose versus Lineage A Naïve, Two Vaccine Doses	****	<0.0001
Lineage A Naïve, One Vaccine Dose versus B.1.351 Prior SARS-CoV-2, Two Vaccine Doses	ns	>0.9999
Lineage A Naïve, One Vaccine Dose versus B.1.351 Naïve, Two Vaccine Doses	****	<0.0001
Lineage A Naïve, One Vaccine Dose versus P.1 Prior SARS-CoV-2, Two Vaccine Doses	ns	>0.9999
Lineage A Naïve, One Vaccine Dose versus P.1 Naïve, Two Vaccine Doses	****	<0.0001
B.1.351 Prior SARS-CoV-2, One Vaccine Dose versus B.1.351 Naïve, One Vaccine Dose	ns	>0.9999
B.1.351 Prior SARS-CoV-2, One Vaccine Dose versus P.1 Prior SARS-CoV-2, One Vaccine Dose	**	0.0099

B.1.351 Prior SARS-CoV-2, One Vaccine Dose versus P.1 Naïve, One Vaccine Dose	ns	>0.9999
B.1.351 Prior SARS-CoV-2, One Vaccine Dose versus Lineage A Prior SARS-CoV-2, Two Vaccine Doses	****	<0.0001
B.1.351 Prior SARS-CoV-2, One Vaccine Dose versus Lineage A Naïve, Two Vaccine Doses	ns	>0.9999
B.1.351 Prior SARS-CoV-2, One Vaccine Dose versus B.1.351 Prior SARS-CoV-2, Two Vaccine Doses	****	<0.0001
B.1.351 Prior SARS-CoV-2, One Vaccine Dose versus B.1.351 Naïve, Two Vaccine Doses	ns	>0.9999
B.1.351 Prior SARS-CoV-2, One Vaccine Dose versus P.1 Prior SARS-CoV-2, Two Vaccine Doses	****	<0.0001
B.1.351 Prior SARS-CoV-2, One Vaccine Dose versus P.1 Naïve, Two Vaccine Doses	ns	>0.9999
B.1.351 Naïve, One Vaccine Dose versus P.1 Prior SARS-CoV-2, One Vaccine Dose	ns	>0.9999
B.1.351 Naïve, One Vaccine Dose versus P.1 Naïve, One Vaccine Dose	****	<0.0001
B.1.351 Naïve, One Vaccine Dose versus Lineage A Prior SARS-CoV-2, Two Vaccine Doses	ns	>0.9999
B.1.351 Naïve, One Vaccine Dose versus Lineage A Naïve, Two Vaccine Doses	****	<0.0001
B.1.351 Naïve, One Vaccine Dose versus B.1.351 Prior SARS-CoV-2, Two Vaccine Doses	ns	>0.9999
B.1.351 Naïve, One Vaccine Dose versus B.1.351 Naïve, Two Vaccine Doses	****	<0.0001
B.1.351 Naïve, One Vaccine Dose versus P.1 Prior SARS-CoV-2, Two Vaccine Doses	ns	>0.9999
B.1.351 Naïve, One Vaccine Dose versus P.1 Naïve, Two Vaccine Doses	****	<0.0001
P.1 Prior SARS-CoV-2, One Vaccine Dose versus P.1 Naïve, One Vaccine Dose	ns	>0.9999
P.1 Prior SARS-CoV-2, One Vaccine Dose versus Lineage A Prior SARS-CoV-2, Two Vaccine Doses	****	<0.0001
P.1 Prior SARS-CoV-2, One Vaccine Dose versus Lineage A Naïve, Two Vaccine Doses	ns	>0.9999
P.1 Prior SARS-CoV-2, One Vaccine Dose versus B.1.351 Prior SARS-CoV-2, Two Vaccine Doses	****	<0.0001
P.1 Prior SARS-CoV-2, One Vaccine Dose versus B.1.351 Naïve, Two Vaccine Doses	ns	>0.9999
P.1 Prior SARS-CoV-2, One Vaccine Dose versus P.1 Prior SARS-CoV-2, Two Vaccine Doses	****	<0.0001
P.1 Prior SARS-CoV-2, One Vaccine Dose versus P.1 Naïve, Two Vaccine Doses	ns	>0.9999
P.1 Naïve, One Vaccine Dose versus Lineage A Prior SARS-CoV-2, Two Vaccine Doses	ns	>0.9999
P.1 Naïve, One Vaccine Dose versus Lineage A Naïve, Two Vaccine Doses	****	<0.0001
P.1 Naïve, One Vaccine Dose versus B.1.351 Prior SARS-CoV-2, Two Vaccine Doses	ns	>0.9999
P.1 Naïve, One Vaccine Dose versus B.1.351 Naïve, Two Vaccine Doses	****	<0.0001
P.1 Naïve, One Vaccine Dose versus P.1 Prior SARS-CoV-2, Two Vaccine Doses	ns	>0.9999
P.1 Naïve, One Vaccine Dose versus P.1 Naïve, Two Vaccine Doses	****	<0.0001

Lineage A Prior SARS-CoV-2, Two Vaccine Doses versus Lineage A Naïve, Two Vaccine Doses	ns	>0.9999
Lineage A Prior SARS-CoV-2, Two Vaccine Doses versus B.1.351 Prior SARS-CoV-2, Two Vaccine Doses	ns	0.8392
Lineage A Prior SARS-CoV-2, Two Vaccine Doses versus B.1.351 Naïve, Two Vaccine Doses	ns	>0.9999
Lineage A Prior SARS-CoV-2, Two Vaccine Doses versus P.1 Prior SARS-CoV-2, Two Vaccine Doses	ns	>0.9999
Lineage A Prior SARS-CoV-2, Two Vaccine Doses versus P.1 Naïve, Two Vaccine Doses	ns	>0.9999
Lineage A Naïve, Two Vaccine Doses versus B.1.351 Prior SARS-CoV-2, Two Vaccine Doses	ns	>0.9999
Lineage A Naïve, Two Vaccine Doses versus B.1.351 Naïve, Two Vaccine Doses	*	0.0345
Lineage A Naïve, Two Vaccine Doses versus P.1 Prior SARS-CoV-2, Two Vaccine Doses	ns	>0.9999
Lineage A Naïve, Two Vaccine Doses versus P.1 Naïve, Two Vaccine Doses	ns	>0.9999
B.1.351 Prior SARS-CoV-2, Two Vaccine Doses versus B.1.351 Naïve, Two Vaccine Doses	ns	>0.9999
B.1.351 Prior SARS-CoV-2, Two Vaccine Doses versus P.1 Prior SARS-CoV-2, Two Vaccine Doses	ns	0.976
B.1.351 Prior SARS-CoV-2, Two Vaccine Doses versus P.1 Naïve, Two Vaccine Doses	ns	>0.9999
B.1.351 Naïve, Two Vaccine Doses versus P.1 Prior SARS-CoV-2, Two Vaccine Doses	ns	>0.9999
B.1.351 Naïve, Two Vaccine Doses versus P.1 Naïve, Two Vaccine Doses	*	0.0302
P.1 Prior SARS-CoV-2, Two Vaccine Doses versus P.1 Naïve, Two Vaccine Doses	ns	>0.9999
